# Charcoal production in the Mopane woodlands of Mozambique: what are the trade-offs with other ecosystem services?

**DOI:** 10.1098/rstb.2015.0315

**Published:** 2016-09-19

**Authors:** Emily Woollen, Casey M. Ryan, Sophia Baumert, Frank Vollmer, Isla Grundy, Janet Fisher, Jone Fernando, Ana Luz, Natasha Ribeiro, Sá N. Lisboa

**Affiliations:** 1 School of GeoSciences, The University of Edinburgh, Edinburgh, UK; 2 Faculty of Agronomy and Forest Engineering, Universidade Eduardo Mondlane, Maputo, Mozambique; 3 Department of Biological Sciences, University of Zimbabwe, Harare, Zimbabwe; 4 Ce3C—Centre for Ecology, Evolution and Environmental Changes, Universidade de Lisboa, Lisboa, Portugal

**Keywords:** African woodland, ecological production function, land cover, woodland structure, non-timber forest products

## Abstract

African woodlands form a major part of the tropical grassy biome and support the livelihoods of millions of rural and urban people. Charcoal production in particular is a major economic activity, but its impact on other ecosystem services is little studied. To address this, our study collected biophysical and social datasets, which were combined in ecological production functions, to assess ecosystem service provision and its change under different charcoal production scenarios in Gaza Province, southern Mozambique. We found that villages with longer histories of charcoal production had experienced declines in wood suitable for charcoal, firewood and construction, and tended to have lower perceived availabilities of these services. Scenarios of future charcoal impacts indicated that firewood and woody construction services were likely to trade-off with charcoal production. However, even under the most extreme charcoal scenario, these services were not completely lost. Other provisioning services, such as wild food, medicinal plants and grass, were largely unaffected by charcoal production. To reduce the future impacts of charcoal production, producers must avoid increased intensification of charcoal extraction by avoiding the expansion of species and sizes of trees used for charcoal production. This is a major challenge to land managers and policymakers in the area.

This article is part of the themed issue ‘Tropical grassy biomes: linking ecology, human use and conservation’.

## 1. Introduction

African savannahs, including the Mopane woodlands that are the focus of this study, are characterized by discontinuous tree cover and a continuous C_4_ grass layer [1]. Woodlands, which are at the more wooded end of the savannah spectrum [2], constitute a major part of savannahs on the continent, covering an estimated 34% of vegetated Africa [3], with Mopane woodlands covering 555 000 km^2^ of southern Africa [4]. Woodlands are dynamic systems, driven by several environmental and human disturbances, both long standing and novel [5]. They hold a unique and diverse flora [6], and are also social woodlands, with millions of rural and urban people relying on them to provide ecosystem services (ES) and livelihood benefits [7–12]. Reconciling the needs of the inhabitants with the need for conservation and the provision of global ES remains a challenge. Woodlands across Africa are changing due to altered disturbance patterns, driven by several social and environmental processes [13,14]. The changing woodlands are likely to affect their ability to provide essential ES, and create trade-offs between different services [12]. African woodlands are multifunctional, diverse and spatially complex systems [5,10], and woodland supply of ES and changes to these are likely to be context-specific, depending on local scale biophysical and social factors.

One of the drivers of woodland change in southern Africa is the charcoal industry. Most southern African countries are engaged in charcoal production, with a value of around 2–3% of GDP [15]. Charcoal is primarily supplied from rural areas and provides affordable energy to 70–90% of the urban population, as well as income-generating opportunities in rural areas [15]. The process of charcoal production can reduce standing woody biomass through selective harvesting of trees, which is the prevalent practice in most African woodlands [16,17]. Clear-cutting for charcoal can occur, particularly on the ‘frontier’ of charcoal production around large cities, where harvesting rates can greatly exceed regrowth [18,19]. Charcoal production is thus likely to impact the woodland resource base, and there may be trade-offs between charcoal production and other ES from woodlands. However, a recent review concluded that few studies have assessed the links between charcoal production and other ES in African woodlands [16], and little is known of the impacts of this large-scale industry on the ability of woodlands to provide other important ES.

To address this need, a large-scale interdisciplinary study, Abrupt Changes in Ecosystem Services and Wellbeing in Mozambican woodlands (ACES, www.miomboaces.wordpress.com), was conducted in the Mabalane District of Gaza Province, southern Mozambique. Mozambique retains large areas of woodlands, which cover 51% of the total land area [20], but with rapid changes occurring [21]. Wood fuels account for 81% of energy consumption in Mozambique [18], with charcoal the dominant fuel in urban centres [22]. The charcoal trade provides employment for millions, supporting more than 5% of the country's population [23]. The full extent of charcoal production and its impacts on woodland resources and potential trade-offs remain largely unknown, mainly due to the sparse data on all aspects of the (largely informal) charcoal industry. Gaza Province is one of the main current supply areas of charcoal to Maputo, and Mabalane District currently has the highest number of licences for charcoal production of any district in Gaza [24].

The aim of this paper is to identify current and likely future trade-offs between charcoal production and the supply of other provisioning ES from woodlands in several villages across the Mabalane District. This has important implications for both the management of woodlands for multiple services, and for livelihoods of local populations. Woodlands provide a multitude of ES [12], and to include them all is beyond the scope of this study. Therefore, the ES investigated were limited to provisioning services that were (i) locally relevant, (ii) were likely to be affected by charcoal production and (iii) could be linked to woodland structure. The social impacts of charcoal production, particularly with regard to benefit distribution, are analysed and debated in a separate publication [25].

Using a combination of biophysical and social datasets, we are able to (i) define locally relevant provisioning ES, (ii) determine how changes in woodland structure might affect services from woodlands and (iii) identify trade-offs of charcoal production with other provisioning services. This study is novel in that it presents one of the first assessments of charcoal trade-offs with other provisioning services from an African woodland. The study also disaggregates woodlands into more realistic complex socio-ecological systems that are not assumed to be uniform in their structure, their use by people or their ability to provide ES, increasing the resolution to local scales; it is at local scales that the impacts of charcoal production are likely to be most important.

## 2. Material and methods

### (a) Methodological approach

Biophysical data were collected to characterize woodland structure, and land cover maps were produced to scale plot level data to the village landscape. We used a combination of household surveys, focus group discussions and key informant interviews to obtain information on local uses of provisioning ES from woodlands and their relative importance, and assess how these services were related to woodland structure. The biophysical and social datasets were combined in ecological production functions to estimate the provision of services from woodlands.

To assess trade-offs of charcoal production, we compare our results between several villages along a chronosequence of charcoal production to determine how provisioning services have changed. The chronosequence includes villages that have already passed their charcoal production peak and then declined production over the last 10 years, to villages that have not yet engaged in charcoal production. We also use scenarios of charcoal production to model potential changes to provisioning services under two different scenarios: one where all previously cut charcoal trees are modelled as intact, and one where all charcoal trees are modelled as cut. Both approaches serve to assess charcoal production trade-offs with other provisioning services, where one approach provides an assessment of current trade-offs, and the scenario approach provides an assessment of likely trade-offs both in the past and in the future.

### (b) Study site

**Figure 1.**
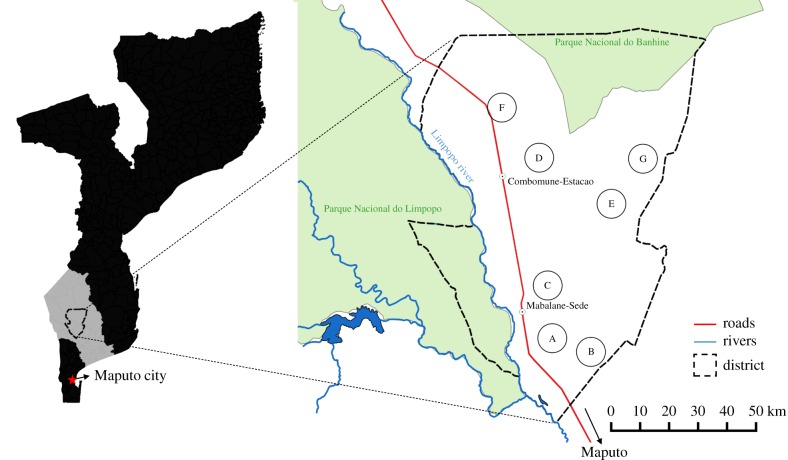
Mabalane District, Gaza Province in southern Mozambique. All study villages (A–G) and their 5 km radii (78.5 km^2^) sample areas are shown. To maintain anonymity of the villages investigated, the villages are represented by letters and their locations are inaccurate.

This study took place from May–October 2014 in the Mabalane District, Gaza Province in southern Mozambique. Mabalane District lies adjacent to the Limpopo River and Limpopo and Banhine National Parks (figure 1), approximately 300 km north of Maputo City. Mabalane District is characterized as an area of dry tropical woodland, consisting mainly of Mopane woodlands interspersed with Combretum and Boscia dominated woodlands, with a C_4_ grass layer. Most of the charcoal produced in Gaza comes from Mopane woodlands, which are dominated by the tree species *Colophospermum mopane*, a dense hardwood species, which produces high-quality, slow-burning charcoal. Mabalane-sede is the district capital situated to the southeast of the district, which can be reached from Maputo by the only (partially) tarred road in the district. Several villages are interspersed throughout the district, with limited access along seasonally passable dirt roads. Low-intensity subsistence agriculture is prevalent, with maize being the dominant crop and livestock rearing under communal grazing systems common. Shangaan is the main language spoken. The area receives a mean annual precipitation of 505 mm, with average annual temperatures of 24°C, with a marked wet season between October and April when 92% of annual precipitation falls (WorldClim dataset, [26]). Soils are classed as loamy sand (82% sand, 13% silt, 5% clay), with a low carbon and nutrient content (0.4% C, 0.05% N).

### (c) Village selection and the charcoal production chronosequence

Across Mabalane District, seven villages with similar climatic conditions, vegetation types and infrastructure, but different stages of charcoal production, were selected for this study. Different charcoal production stages were determined according to the method of Baumert *et al.* [25], using the classification criteria: (i) current number of charcoal licence holders in the community land, (ii) production quantity of licensed charcoal and (iii) year with highest charcoal production according to village narratives. For this study, this translated into a charcoal production chronosequence of villages with little or no charcoal production (classified as *pre-boom* villages), to those experiencing a charcoal production peak (classified as *boom* villages) and to villages where the peak had already passed (classified as *post-boom* villages) at the time of this study.

Thereby, the villages were labelled A–G in order of charcoal production stages (figure 1). Villages A, B and C were classified as *post-boom* villages as they had already passed their charcoal production peaks in 2006, 2009 and 2013, respectively, after which a decline in large Mopane trees saw large-scale charcoal production operators withdrawing to new areas for exploitation. Villages D and E were classified as *boom* villages, as in these villages large-scale charcoal production started in 2011, and was still at high levels in 2014 at the time of the study. Villages F and G were classified as *pre-boom* villages either because large-scale charcoal production had not yet begun and remained at small-scales (village F), or charcoal production had not yet begun (village G).

### (d) Biophysical data collection

#### (i) Woodland structure

Woodland structure in each village was assessed using standard forest inventory methods. Forest inventories were conducted within a sampling area of 5 km radius (78.5 km^2^) from the centre of each village (figure 1). The centre was defined as the point where the community meeting house was located. A 5 km radius was chosen as this area was deemed representative of immediate village landscape resources within a reasonable daily walking distance from households. Furthermore, a 5 km buffer minimized outside influences from neighbouring villages, but some overlaps with neighbouring villages was observed.

Within each village sampling area, 24 circular forest plots (20 m radius) were measured, selected from several hundred randomly placed plots using a random number generator. The final selection was based on those plots that were found not to be in permanently waterlogged areas, in active agricultural fields, or in built up areas. Village D was the pilot study site, where the plot design differed in that plots varied in size and had nested plot designs. Only 19 plots were measured in village D. The pilot village was included in this study as the methods and outputs were robust and comparable with the methods and outputs in the other villages. From the total forest plot dataset, nine plots were excluded from analyses due to errors in measurements or missing data (total plots *n* = 154).

Within each plot, for each stem more than 5 cm diameter-at-breast-height (DBH, 1.3 m from ground), local species name, point of measurement and condition of each tree stem (live or dead, cut or broken) were recorded, with help from villagers knowledgeable about the flora. The remnant stumps of all cut stems regardless of diameter were included in inventories, where the height of the diameter measurement was recorded as well as total stump height. To adjust tree stem diameters measured at less than 1.3 m, a correction function was used to estimate the DBH at 1.3 m (see electronic supplementary materials). Above-ground woody biomass (AGB, Mg C ha^−1^) of each plot was estimated using measured or estimated DBH measurements of tree stems and the allometric equation from Ryan *et al.* [27], as this equation showed close agreement with other relevant allometric equations [28,29] and was deemed the most suitable based on location and measurement methods. Local tree names were identified to species where possible using species identification keys [30,31]. Samples of trees taken in the field were also identified for cross-validation by botanists at the Universidade Eduardo Mondlane in Maputo. As species identification was not possible for all local names, all analyses were conducted using local tree names.

Grass biomass was collected and weighed within 1 m^2^ quadrats placed at 10 m distances from the centre of the plot in each cardinal direction (*n* = 4 per plot). Grass sub-samples were taken and dried in an oven at 70°C for 48 h, and dry weight was determined. The dry weight fraction was used to estimate dry grass biomass and mean dry grass biomass determined (Mg ha^−1^). Recently burnt plots were not included in this analysis. The composition of grass biomass by species was not possible to determine due to difficulties of identification during the dry season.

Coarse woody debris was measured using the methods outlined by Waddell *et al.* [32], along four 20 m transects from the centre to the edge of each plot in each cardinal direction. The diameter of each woody piece encountered along each transect, its length, the decay class and the local tree species name was recorded. Coarse woody debris included all woody pieces with more than 3 cm diameter at intersection and more than 0.5 m length. Total biomass of coarse woody debris (Mg C ha^−1^) within each plot was calculated following Waddell *et al.* [32].

#### (ii) Land cover classification and biomass mapping

Supervised land cover mapping was undertaken using a combination of Landsat 8 and ALOS PALSAR remote sensing products, and ground control points based on both the plot data and other observations. The classification legend for woodland types was developed using a hierarchical clustering of the plot data (based on the Bray–Curtis dissimilarities and the average linkage method). All calculations were done using the Vegan v. 2.0.10 package [33] in R statistical software [34]. Dissimilarities between plots were calculated based on relative abundance of AGB of each species. Distinct clusters were identified using the Calinksi–Harabasz criterion implemented with k-means clustering in the cascadeKM function in Vegan. Each plot was then classified into one of the identified clusters (i.e. woodland types). All other non-woodland land cover classes were lumped together as ‘other’.

The land cover map was based on the classification of multi temporal Landsat 8 data (images from May and October 2014) and ALOS PALSAR 2 HV backscatter (October/November 2014). The classification was created using a Support Vector Machine classifier implemented in ENVI v. 5.2 (Exelis Visual Information Solutions, Boulder, Colorado) using 430 polygons of ground data based on our observations and forest plot data (collected as described above). Twenty-five per cent of the ground data was set aside and used for validation purposes. The classification had an overall accuracy of 87% (Kappa coefficient 0.8) and was effective at distinguishing different floristic types of woodland. Among woodland types, the two dominant classes (Mopane and Combretum woodlands) were easily distinguished with a seperability of 1.9–1.99, whereas the less dominant classes had a seperability of 1.1.

We used a biomass map constructed from the ALOS PALSAR 2 data obtained in November 2014. Images were calibrated, terrain corrected and de-speckeled using SNAP v. 2.0 and exported at 15 m pixel size. The image was georeferenced to a mosaicked, pansharpened Landsat 8 image (Sept 2014) using ENVI v. 4.8. HV backscatter was used to estimate above-ground woody biomass (Mg C ha^−1^) of the Mabalane District at 15 m pixel resolutions following the regression of Ryan *et al.* [21].

### (e) Social data collection

In each of the seven sampled villages, a household list was compiled based on the definition of households as people ‘eating from the same pot’. Households were then randomly selected for a socio-economic household survey. Household surveys were used in this study to identify the key provisioning services from woodlands that were most commonly used by local people. Focus group discussions and semi-structured interviews [35] were conducted with village leaders, community groups, charcoal producers and traditional healers, to gain information on how the key provisioning services identified from the household survey were related to woodland structure and composition. This was determined by asking what particular woodland tree or grass species (recorded using local names for plants) and other plant characteristics were used or preferred by the local population for each of the provisioning services, determined from discussions and interviews. Linking provisioning ES to woodland tree or grass species precludes the assessment of services not provided by trees or grasses in this study. It was assumed that if a specific use for a particular species was recorded in one location or by one individual, it applied to the whole study area. It is recognized that not all the different species or characteristics of woodland plants relating to each service were recorded, so we refer to the recorded uses as ‘known species uses’.

Perceived temporal changes to provisioning ES in each village were recorded using a trend analysis (one of a suite of methods known as participatory rural appraisal [36]). This involved asking a focus group of key informants in each village about temporal changes in abundance and access to resources from woodlands, from the year villagers returned to their villages after the civil war (1994/1995) until the present day, indicating a general direction of change over time. Specific provisioning ES were not asked about directly, but the discussion was guided by asking about changes to any provisioning ES from woodlands. If a particular ES was not mentioned it was assumed it was not important or perceived as changing.

### (f) Quantifying ecosystem service provision and trade-offs

We estimate the available ES provided by woodlands using the ecological production function approach [37,38]. Production functions define how changes in an ecosystem's structure or function are likely to affect flows of ES from those ecosystems. The functions combine our biophysical and social datasets to account for both service supply, and the preferences of people who use these services. All service provision was determined as a function of woodland structure (e.g. above-ground biomass, stem density, stem size distribution and species composition) assuming that changes to woodland structures affect changes in service provision. In this study, service provision is expressed in biophysical terms (e.g. tons of biomass) for each study village (see the electronic supplementary material for the production function equations).

Scenarios of charcoal production were used to simulate changes to woodland structure and subsequent changes in provision of ES from woodlands. Two scenarios were compared with the current state of woodlands. In Scenario 1, the ‘no charcoal’ scenario, we assumed no trees had been used for charcoal in the past—all the observed cut stems of trees suitable for charcoal production were modelled as intact. In Scenario 2, ‘total charcoal’, all trees suitable for charcoal were modelled as cut. The modelled changes to woodland structure were then used to calculate ES provision under the different scenarios within each village using the ecological production functions. Changes to the estimated provisioning services for each scenario were then compared with the current estimates to assess likely impacts of charcoal production in biophysical terms. The ‘no charcoal’ scenario serves as a way of determining what past trade-offs of charcoal production are likely to have been, and the ‘total charcoal’ scenario the possible future trade-offs of charcoal production. The scenarios model selective logging for charcoal production, rather than clear felling, as selective logging is the prevalent form of wood extraction for charcoal production in Africa [16,17], and is representative of current practices in the study area.

Standard errors of the mean at the plot level were propagated to the total estimated provision of ES at the village landscape scale, both for current and scenario estimates, using standard error propagation formulae and assuming independence. All errors on estimates are presented as 95% confidence intervals.

## 3. Results and analysis

### (a) Ecosystem services and links to woodland structure

The most commonly used provisioning services from woodlands were charcoal, firewood, woody construction materials, thatching grass, food, medicinal plants and livestock forage (table 1). The household survey sampled more than 80% of all households in the sample villages. Of the sampled households, more than 70% produced charcoal within the last 12 months, but with varying prevalence between villages (table 1). Charcoal was primarily sourced from local woodlands, but occasional sources included trees cut when creating new agricultural fields. Firewood was commonly used in all villages, with 86–100% of households using firewood as their primary source of fuel in the past 12 months (table 1). Of these, 71% collected firewood from woodlands whereas the remaining 29% collected firewood from agricultural fields or fallows. The use of woody construction materials for building of houses was also commonly reported (61%), whereas use of grasses, primarily for roof thatch, was less common (35%, table 1). Collection of food from woodlands was undertaken by 19% of households, and no village had more than 28% of households using this service in the past 12 months (table 1). Fruits were the primary food collected (98%). Medicinal plants were not commonly used over the past 12 months (15%, table 1) but were mentioned as an important alternative when pharmaceutical medicines were not available or were unaffordable. Livestock rearing was prevalent for all villages (more than 60%), and 45% of households that owned livestock used woodlands for pasture or foraging for their livestock, at least on occasion over the past year (table 1). Other provisioning services derived from woodland plant materials were recorded, such as furniture and tool making or baskets and mats, but less than 5% of households reported active engagement in production of these products in the past 12 months, and these were not considered further.

**Table 1.** The number of households (HH) and the per cent (%) of sampled households using provisioning ecosystem services from woodlands in each village.

village	post-boom			boom		pre-boom		total
	A	B	C	D	E	F	G	
total number of HH in village, *N*	38	29	63	42	58	55	27	312
HH sampled, *n* (% of *N*)	34 (90%)	25 (86%)	51 (81%)	36 (86%)	42 (72%)	48 (87%)	24 (89%)	260 (83%)
HH producing charcoal (% of *n*)	29 (85%)	22 (88%)	46 (90%)	23 (64%)	21 (50%)	42 (88%)	0 (0%)	183 (70%)
HH using firewood as primary fuel for cooking (% of *n*)	33 (97%)	25 (100%)	49 (96%)	31 (86%)	42 (100%)	48 (100%)	24 (100%)	252 (97%)
HH using woody materials for construction of houses (% of *n*)	19 (56%)	18 (72%)	25 (49%)	21 (58%)	24 (57%)	38 (79%)	14 (58%)	159 (61%)
HH using grass for construction of houses (% of *n*)	9 (26%)	12 (48%)	30 (59%)	18 (50%)	5 (12%)	9 (19%)	7 (29%)	90 (35%)
HH collecting food from woodlands (% of *n*)	9 (26%)	3 (12%)	10 (20%)	10 (28%)	4 (10%)	6 (13%)	6 (25%)	48 (19%)
HH using medicinal plants (% of *n*)	2 (6%)	5 (20%)	11 (22%)	7 (19%)	5 (12%)	6 (13%)	4 (17%)	40 (15%)
HH whose livestock forage in woodlands (% of *n*)	12 (35%)	15 (60%)	12 (24%)	5 (14%)	27 (64%)	29 (60%)	16 (67%)	116 (45%)

Therefore, the provisioning services from woodlands included in this study were grouped as charcoal, firewood, woody construction materials, food, medicinal plants and grass. Each service was linked to woodland structural data by ascertaining which size and species of plants from the woodland were used for each, informed by the focus group discussions and key informant interviews. For those services provided by trees, the service provision was linked to local names of trees (electronic supplementary material, table S1). Services from grasses could not be related to specific grass species or characteristics, as grass species abundance data for woodlands was not available. Therefore, we use a relationship between measured grass biomass and stem density (electronic supplementary material, figure S1) to estimate maximum potential grass biomass, and use this as a proxy for the availability of grass-related services.

The number of tree species that provided each service varied from five to 39 (electronic supplementary material, table S1). Charcoal and firewood had the least number of tree species used (six and five species used, respectively), indicating highly selective species preferences for these services. Species used for firewood also overlapped with those used for charcoal, where three out of the five species were shared. There was a strong preference for *C. mopane* species for charcoal production in the study area, and *C. mopane* was also the only species listed that could be used for all five services related to trees (electronic supplementary material, table S1). From plot data, *C. mopane* had the highest number of recorded cut stems (electronic supplementary material, table S2), indicating this species is heavily used and extracted. Woody construction materials were more diverse, with 10 known tree species used for this service. Food and medicinal services were the most diverse and least selective of all the services (21 and 39 tree species used, respectively).

### (b) Land cover classification and woodland structure

Five different vegetation types were identified across the study area from the hierarchical cluster analysis: Androstachys forest, Mopane woodland, Combretum woodland, Boscia woodland and shrub Mopane (table 2). Androstachys forest was characterized by the dominance of *Androstachys johnsonii*, where stem density and AGB was on average 1764 ± 116 stems ha^−1^ (±s.e.m.) and 31.7 ± 2.5 Mg C ha^−1^ (table 2); there was almost no grass biomass (0.06 ± 0.02 Mg ha^−1^), hence it was characterized as a forest rather than a woodland. Androstachys forest had the greatest mass of coarse woody debris (3.57 ± 0.63 Mg C ha^−1^), due to many broken or dead stems, and occurred as patches in the landscape interspersed among other land cover types. Mopane woodlands had lower stem density and AGB (means of 769 ± 65 stems ha^−1^ and 11.8 ± 1.6 Mg C ha^−1^), but greater grass biomass (0.66 ± 0.13 Mg ha^−1^) than Androstachys forest, and were dominated by *C. mopane*. Combretum woodlands were similar to Mopane woodlands in their structure, but had greater grass biomass (1.06 ± 0.13 Mg ha^−1^) and tree species diversity than Mopane woodlands. Combretum woodlands were the most diverse of all the woodland types (species richness 7.1 ± 0.5 and evenness of 0.66 ± 0.03), but *Combretum* spp. dominated. Boscia woodlands were characterized by the presence and dominance of *Boscia albitrunca*. Boscia woodlands had similar stem densities to Combretum woodlands, but with much less AGB (5.4 ± 1.38 Mg C ha^−1^) due to smaller stem sizes. The shrub Mopane woodland was characterized by the presence of *Aloe* spp. and small (less than 2 m height) *C. mopane* trees, the only two species occurring in this woodland type. The stem density and AGB were low (103 stems ha^−1^ and 7.31 Mg C ha^−1^) due to the small stature of most of the Mopane trees (below the 1.3 m measurement height). Grass biomass was also low in this woodland type (0.34 Mg ha^−1^), despite the relatively open canopy. For a visual comparison between woodland types see supplementary materials, figure S2.

**Table 2.** Characteristics of each woodland type based on plot data across all villages. Mean stem density, above-ground woody biomass, dry grass biomass and coarse woody debris are shown. Mean plot level diversity measures of tree species richness and evenness do not include plots from village D. Errors are standard errors of the mean. Errors could not be calculated for shrub Mopane as *n* < 4.

woodland type	indicator species	*N*	stem density (stems ha^−1^)	above-ground biomass (Mg C ha^−1^)	grass biomass (Mg ha^−1^)	coarse woody debris (Mg C ha^−1^)	species richness	species evenness (index)
Androstachys forest	*Androstachys johnsonii*	24	1764 ± 116	31.7 ± 2.5	0.06 ± 0.02	3.57 ± 0.63	6.6 ± 1.0	0.22 ± 0.05
Mopane woodland	*Colophospermum mopane*	51	769 ± 65	11.8 ± 1.6	0.66 ± 0.13	0.90 ± 0.21	5.3 ± 0.4	0.50 ± 0.03
Combretum woodland	*Combretum* spp.	63	639 ± 52	12.8 ± 1.4	1.06 ± 0.13	0.98 ± 0.37	7.1 ± 0.5	0.66 ± 0.03
Boscia woodland	*Boscia albitrunca*	13	582 ± 78	5.4 ± 1.38	0.79 ± 0.22	0.72 ± 0.22	3.1 ± 0.5	0.31 ± 0.08
shrub Mopane	*Aloe* spp.*, Colophospermum mopane*	3	103	7.31	0.34	0.02	2.0	0.34

**Figure 2.**
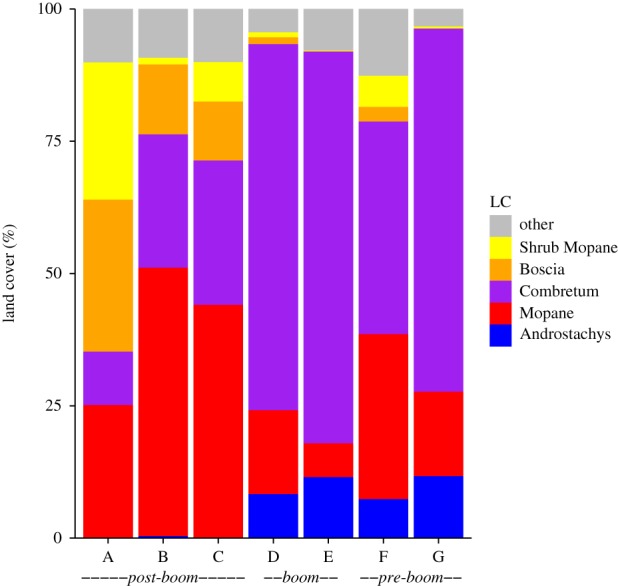
Land cover (%) for each village landscape within a 5 km radius (78.5 km^2^) of village centres.

Land cover composition varied between villages (figure 2) but woodlands dominated all village landscapes. Most villages were dominated by Mopane and Combretum woodlands; only village A had a greater proportion of Boscia and shrub Mopane woodlands, which were not extensive in other villages. Villages to the south of the study site (villages B–C) had greater proportions of Mopane woodlands, and villages to the north (villages D–G) had greater proportions of Combretum woodland. Androstachys forest only occurred in those villages located to the north of the study site (villages D–G).

Owing to the post hoc classification of forest plots, the distribution of plots within woodland types was not always proportionate to the village land cover (table 3). In some villages, few or no plots fell within certain woodland types despite having more than 5% of land area of that type (figure 2). Therefore, plots were amalgamated within chronosequence classes (*post-boom, boom*, *pre-boom*) to increase the sample size for each woodland type within a class. However, even when plots were amalgamated only three plots fell within the shrub Mopane class for the entire dataset, and for the *boom* villages (D–E) only two plots fell within Mopane woodlands. Therefore, some caution is required when upscaling plot data to the landscape level for shrub Mopane woodlands in all villages, and Mopane woodlands in *boom* villages, as error estimation was not possible when *n* < 4.

**Table 3.** Number of sample plots within each village and woodland type based on post hoc classification from the land cover map.

class	village	Androstachys	Mopane	Combretum	Boscia	shrub Mopane	total
post-boom	A	0	3	4	13	3	23
	B	0	19	4	0	0	23
	C	0	11	9	0	0	20
	total	0	33	17	13	3	
boom	D	6	1	12	0	0	19
	E	5	1	17	0	0	23
	total	11	2	29	0	0	
pre-boom	F	7	14	2	0	0	23
	G	6	2	15	0	0	23
	total	13	16	17	0	0	
total		24	51	63	13	3	154

### (c) Ecosystem services availability at village scales

**Figure 3.**
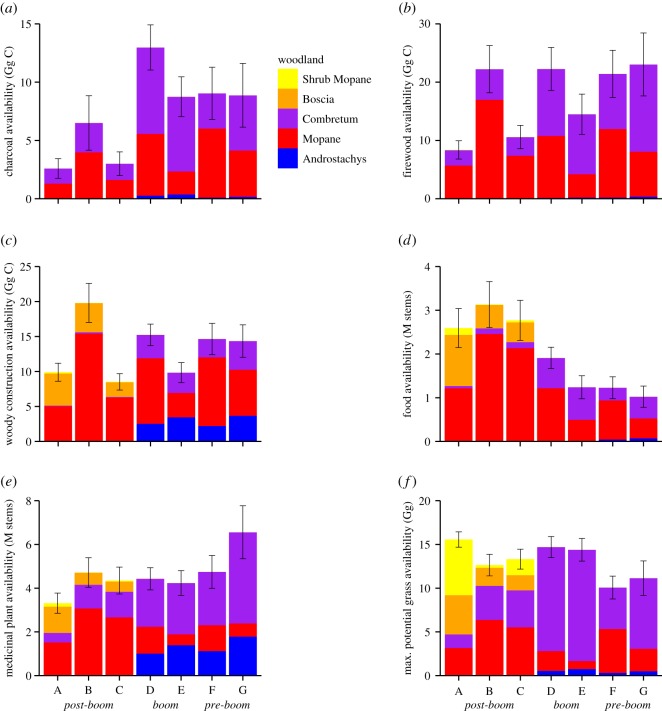
Current estimated ecosystem service availability of (*a*) charcoal, (*b*) firewood, (*c*) woody construction materials, (*d*) wild food, (*e*) medicinal plants and (*f*) estimated maximum potential for grass, with the proportion provided by each woodland type within the village sample areas shown. Error bars are 95% confidence intervals.

Total current ES availability for each village was estimated by applying the production functions to each village landscape (figure 3). The production function parameters calculated from plot data were averaged across chronosequence classes (table 3; electronic supplementary material, table S3). We applied the averaged parameters to each individual village within that class. Therefore, differences between villages within a class are due to land cover differences rather than woodland structural differences. However, comparisons between classes still take into account differences in land cover and woodland structure.

The production functions showed that Mopane and Combretum woodlands together provided the highest diversity and quantity of provisioning services in all villages, and are key to the provision of services in the study area (figure 3). All six of the provisioning services were available in each village, but the total provision varied between villages due to a combination of differences in woodland structure and land cover. Charcoal availability was the lowest in *post-boom* villages A and C, and was higher (no overlap in 95% CIs) for *boom* and *pre-boom* villages (figure 3*a*). For all villages, charcoal was provided by Mopane and Combretum woodlands, with small amounts from the Androstachys forests. Firewood was similarly provided from Mopane and Combretum woodlands, where villages A and C had the lowest availability of firewood (figure 3*b*). Woody construction materials were primarily provided by Boscia and Mopane woodlands in the *post-boom* villages to the south, and by Androstachys, Mopane and Combretum woodlands in *boom* and *pre-boom* villages to the north (figure 3*c*). Again, *post-boom* villages A and C were found to have the lowest availability of woody construction materials. Village B had higher availability of charcoal, firewood and woody construction materials in comparison with other *post-boom* villages due to the larger Mopane and Combretum woodland areas (figure 2).

Food availability showed an opposite pattern to charcoal, firewood and woody construction materials, as food was higher for *post-boom* villages (figure 3*d*). Food was primarily provided by Mopane woodlands in all villages, but Boscia woodlands also provided some food services for *post-boom* villages in the south, and Combretum woodlands in *boom* and *pre-boom* villages in the north. Medicinal plants were equally available in villages B–F, but village A had less availability (figure 3*e*). Medicinal plants were primarily provided by Mopane, Combretum and Boscia woodlands in *post-boom* villages to the south, and by Combretum, Androstachys and Mopane woodlands for *boom* and *pre-boom* villages to the north.

The maximum potential for grass biomass, estimated as a function of stem density (electronic supplementary material, figure S1), showed similar potentials across *post-boom* and *boom* villages, but *pre-boom* villages had slightly lower potentials (figure 3*f*). Combretum and Mopane woodlands provided the majority of grass potentials, but in village A, shrub Mopane and Boscia woodlands had the highest potentials for grass. However, this was contrary to our observations (table 2), where shrub Mopane and Boscia woodlands had very low measured grass biomass. Therefore, the modelled maximum potentials for grass biomass are likely to be unrealistic and real availability of grass biomass may be smaller than estimated here.

### (d) Charcoal production trade-offs with other ecosystem services

There was a general decrease in the number of services perceived as declining along the charcoal production chronosequence from villages A to G in trend analyses (table 4). *Post-boom* and *boom* villages A–D had the greatest number of services perceived as declining, whereas *pre-boom* villages F and G had no perceived declines in any of the services. There was a perceived historical decline in charcoal resource availability for all *post-boom* villages A–C and *boom* village D in the trend analyses. In village C, one respondent even mentioned that they tried producing charcoal from alternative tree species (*Combretum* spp.) but that buyers rejected the charcoal in favour of charcoal made from *C. mopane* trees elsewhere, suggesting a scarcity in suitable Mopane charcoal trees. Firewood was only mentioned as declining in villages A and D. Woody construction services were perceived as declining in all *post-boom* and *boom* villages A–E. Food and medicinal plants from woodlands were not perceived as declining in any of the villages. Services related to grass, such as roof thatch and grazing, were not mentioned by any of the villages in the trend analysis, and therefore we assume these services were not changing or were less important.

**Table 4.** Analysis of the temporal trends in provisioning ecosystem services since 1993/1994 as perceived by each village.

class	village	charcoal	firewood	woody construction	food	medicinal plants	grass
post-boom	A	decline	decline	decline	no change	no change	no change
	B	decline	no change	decline	no change	no change	no change
	C	decline	no change	decline	no change	no change	no change
boom	D	decline	decline	decline	no change	no change	no change
	E	no change	no change	decline	no change	no change	no change
pre-boom	F	no change	no change	no change	no change	no change	no change
	G	n.a.	no change	no change	no change	no change	no change

**Figure 4.**
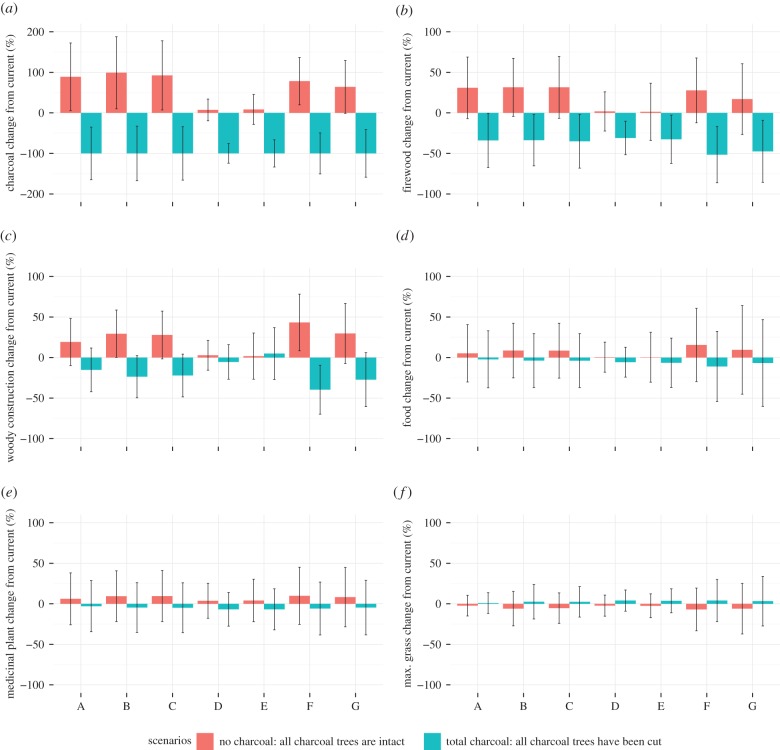
Estimated changes (%) in ecosystem service availability of (*a*) charcoal, (*b*) firewood, (*c*) woody construction materials, (*d*) wild food, (*e*) medicinal plants and (*f*) estimated maximum potential for grass in relation to current availabilities under different charcoal scenarios. The ‘no charcoal’ scenario estimates past ES availability by modelling all suitable charcoal trees as intact (i.e. they had never been cut). The ‘total charcoal’ scenario estimates future ES availability by modelling all suitable charcoal trees as cut. Negative changes are losses and positive changes are gains in ES availability in comparison to current availabilities. Error bars are 95% confidence intervals.

The perceived declines in charcoal resources in *post-boom* villages were corroborated by scenario results. Under the ‘no charcoal’ scenario, where all suitable charcoal trees are modelled as intact (i.e. pre-charcoal extraction), *post-boom* villages A–C had the greatest increases in charcoal availability of 89–99% from current estimates (figure 4*a*; for absolute changes in ES availabilities, see electronic supplementary material, figure S3). *Boom* village D also perceived a decline in charcoal resources, but this was not supported by the ‘no charcoal’ scenario, where *boom* villages D–E only had small increases in charcoal availability (less than 10%) when compared with current levels (figure 4*a*). This was probably due to the small number of plots (*n* = 2, table 3) located in Mopane woodlands in these villages, increasing uncertainty on estimates. Villages F and G also had large increases in charcoal availabilities under the ‘no charcoal’ scenario, despite their lack of perceived changes in charcoal availabilities in trend analyses and their classification as *pre-boom* villages. The ‘no charcoal’ scenario for village F indicated charcoal availability was 78% larger in the past than current estimates (figure 4*a*). Their lack of perceived changes in charcoal availability was probably due to the absolute availability of charcoal being higher than *post-boom* villages (figure 3*a*), and any losses to date may therefore not have been large enough to decrease charcoal availability to scarce levels. Village F was classified as a *pre-boom* village as it had not reached peak charcoal production at the time of this study, and charcoal production was restricted to local small-scale producers. This suggests that even small-scale production can have a measureable impact on charcoal resources. The local population of village G did not produce charcoal, so the loss of charcoal availability between past and present estimates as modelled in the ‘no charcoal’ scenario (figure 4*a*) were probably caused by the effect of amalgamated plot level data for both *pre-boom* villages being applied to village G (table 3). For all villages, the estimated changes in charcoal availability in the future under the ‘total charcoal’ scenario showed a 100% loss of current charcoal availability, as expected, as the ‘total charcoal’ scenario models all suitable charcoal trees as cut or removed from woodlands.

Firewood was only perceived as declining in villages A and D. This was surprising as most of the species used for firewood overlapped with those used for charcoal (electronic supplementary material, table S1), suggesting villages with longer histories of charcoal production should be more likely to see a decline in firewood. The ‘no charcoal’ scenario indicated that firewood availability was higher in the past compared with current estimates for all villages by up to 32% (figure 4*b*), although most of their 95% confidence intervals overlapped with the zero line, suggesting they were not significantly higher than current estimates. Charcoal production may not have impacted on perceived firewood availability to date, and firewood may still be available despite charcoal production if woody residues and smaller charcoal trees that are not targeted for charcoal can still provide suitable firewood, or if alternative species not usually preferred for firewood were used instead. The modelled changes to firewood availability in the future under the ‘total charcoal’ scenario showed decreases in firewood availability for all villages (figure 4*b*). Further charcoal production is therefore likely to decrease firewood availability in future for all villages. However, future losses should not decrease availability by more than 52% of current estimated values but, at the upper limit of confidence bounds, could decrease by up to 86% from current estimates (figure 4*b*). Future decreases are likely to be more severe in villages that have less absolute availability of firewood, such as villages A and C, where further losses would mean few firewood resources remaining in absolute terms (figure 3*b*).

Woody construction services were perceived as declining in all *post-boom* and *boom* villages, but not in *pre-boom* villages. However, the modelled availability of woody construction materials in the ‘no charcoal’ scenario showed higher estimated availabilities in the past when compared with current estimates for all villages by up to 43% (figure 4*c*); low changes in *boom* villages D–E are probably due to the low sample size in Mopane woodlands (table 3). One respondent in *pre-boom* village F said that they did not see a decline in construction materials as they also used *A. johnsonii* for construction purposes, which is not used for charcoal, so there was no conflict between uses. This may explain why *post-boom* villages reported a decline in construction materials, whereas *pre-boom* villages did not, if alternatives to Mopane trees for construction such as Androstachys trees are not available. Diversity of woodland types within a village landscape may therefore contribute towards reducing impacts of charcoal production on ES provision. Furthermore, *pre-boom* villages may not have perceived a loss in woody construction materials if absolute availability of woody construction materials was higher (figure 3*c*). The modelled changes to woody construction materials in the future under the ‘total charcoal’ scenario show that further decreases of woody construction materials in all villages can be expected if charcoal production continues (figure 4*c*). However, woody construction availability should not decrease by more than 40% of current values but, at the upper limit of confidence bounds, woody construction availability could decrease by up to 70% (figure 4*c*). These modelled decreases are likely to be more severe in villages that have less current availability of woody construction materials or less diversity of provisioning woodlands (figure 3*c*), such as villages A and C, where further losses would mean few resources remaining in absolute terms.

Food and medicinal plant availabilities all showed a slight increase from current estimates in the ‘no charcoal’ scenarios, and a slight decrease from current estimates in the ‘total charcoal’ scenarios, but differences to current estimates were small (less than 15%) and unlikely (95 CIs overlapped the zero line) to be different to current estimates (figure 4*d*,*e*). Food and medicinal plants from woodlands were not perceived as declining in any of the villages, perhaps due to their infrequent use (table 1), greater diversity of species used and less overlap with species used for charcoal (electronic supplementary material, table S1). Most villages also mentioned that cutting down of fruit trees was forbidden, so overlaps with other uses, which are more destructive, are unlikely. Furthermore, food and medicinal plant availability was measured in terms of stem density, and impacts of charcoal may therefore be less severe than if measured in terms of biomass, as selective charcoal extraction would remove large trees, decreasing biomass more so than stem density. Stem density may even be increased after charcoal production if coppicing of cut stems or regeneration occurs.

Services related to grass, such as roof thatch and grazing, were not mentioned in any of the village trend analyses, and were therefore assumed not to be changing. The modelled maximum grass biomass potentials all showed a slight decrease in ‘no charcoal’ scenarios and a slight increase in ‘total charcoal’ scenarios (figure 4*f*). However, the differences to current values were small (less than 7%) and unlikely to be different to current estimates (figure 4*f*). If charcoal extraction had a small effect on stem density, maximum potentials for grass biomass would also show small effects, as it was modelled as a function of stem density (electronic supplementary material, figure S1).

## 4. Discussion

### (a) Charcoal production trade-offs

Savannahs are important for providing a multitude of environmental, economic and cultural benefits to millions of rural and urban people worldwide, but despite this, face several conservation threats [39]. The savannahs and woodlands of Africa are also multifunctional ecosystems, and the issue of charcoal production trade-offs with other ES is just a small part of the many management challenges facing these systems [10]. However, understanding the impact of charcoal production and trade-offs with other ES can contribute towards reconciling the needs of the inhabitants with the need for conservation and the provision of ES.

In this study, we find that trade-offs of charcoal production in our study area are likely to be with firewood and woody construction material services from Mopane woodlands. These trade-offs have already been perceived to occur, especially in villages with longer histories of charcoal production, and projected estimates indicate that these trade-offs are likely to increase in future for all sampled villages if charcoal production continues. However, the results also suggest that there is some resilience to the impacts of charcoal production on other ES in our study area. Trade-offs were mediated by village landscape configurations, where greater woodland diversity increased availability of alternatives in some cases. Also, villages with greater absolute availability of services were less likely to perceive declines in services despite large modelled losses. Conversely, villages with landscapes dominated by low-quality woodlands (shrub Mopane and Boscia woodlands) had less resource availability, and were more likely to perceive declines in ES. Impacts of charcoal production are therefore not uniform, and some villages may be more vulnerable to impacts of charcoal production both in the past and in the future.

In the African context, this study suggests that where charcoal extraction occurs on a selective basis, the impact on other provisioning services may be minimal. This is in contrast with the rhetoric on charcoal of the ‘woodfuel crisis’ in the 1970s and 1980s, where woodfuel demand was projected to outstrip supply causing large-scale deforestation. The woodfuel crisis has not materialized [40,41], but there has been some suggestion that there might be a return to it [42], largely due to intense exploitation of woodlands occurring at ‘hot spots’ around large urban centres. However, the ecological evidence is sparse to support the view that charcoal production causes widespread deforestation and severe impacts [16,43,44], and the sustainability of charcoal production is highly context-specific. Concerns over sustainability and impacts of charcoal production at ‘hot spots’ remain well-founded, and management challenges for charcoal production are to avoid the creation of over harvesting in ‘hot spots’, leading to deforestation.

### (b) Implications for management

The woodlands of southern Africa, including Mopane woodlands, are multifunctional and provide a range of services [10,45,46]. To manage Mopane woodlands for multiple ES requires detailed understanding of the processes that govern their ES provision and use by people. This study found that a combination of the woodland structure and floristics ultimately determines the ability of woodlands to provide key services to local populations, and the impacts that charcoal production had on these services. The disaggregation of woodlands into specific woodland types also helped to show that not all woodlands were equally good at providing services, and the quality of the woodland type was key to determining the provision of services. Therefore, assuming simple land cover-ES links, as is often the case in trade-off analyses [47], will not suffice; it was the quality of the land cover that was linked to the provision of many of the key ES in these ecosystems, and their interactions which determined their trade-offs. Thus, gaining a greater understanding of the ecology behind the provision of ES in African woodlands will aid the management of these woodlands for multiple services.

In our study, it was found that several services could be provided from the same woodland type, supporting the view of multifunctional woodlands. However, in order to assess optimal and sustainable management strategies of these woodlands for multiple uses, growth and recovery rates of woodlands from disturbance are needed. Very few studies have been conducted on the fundamental growth rates and recovery of *C. mopane* from disturbances [48–50], but growth rates in our study area are likely to be slow (less than 1 mm radial growth per annum) given the low rainfall and poor soil conditions [51,52]. This has implications for providing sustainable low-impact charcoal from Mopane woodlands, and any forest management strategy should consider that *C. mopane* may take many decades to recover from charcoal harvesting, increasing the area required for sustainable extraction and the timescales over which trade-offs are likely to affect local people. Forest managers should also consider that villages with landscapes dominated by lower quality and less diverse woodland types are likely to be more vulnerable to further charcoal production impacts, and these villages may need to be prioritized in management efforts.

The trade-offs identified in this study are context-specific, in that they are only representative of the current situation in Mabalane District, and may not be representative of future scenarios where the species or characteristics of trees used for charcoal production change. For instance Malimbwi *et al.* [15] show that charcoal production often moves from a highly selective phase to a ‘take anything’ phase as the resource becomes scarce. If this switch did occur it would have very different impacts on ES than those found here, most probably exceeding current trade-offs and impacting several other ES [16]. To maintain ES and avoid further impacts of charcoal production both now and in future, increased intensification (i.e. the ‘take anything’ phase) of charcoal production should be avoided, and the production frontier should continue to expand into other Mopane woodland areas [24]. This strategy would increase the area being degraded, but evidence from this study suggests that selective charcoal extraction does not completely eliminate other provisioning services, and if left to re-grow following charcoal extraction, the woodlands would be able to recover more quickly than if intensive extraction had resulted in largely deforested areas or conversion to other intensive land uses [48]. There is some evidence that this strategy is already occurring in neighbouring districts further to the south, where charcoal extraction is currently banned due to overexploitation, encouraging the charcoal frontier to continue moving to new areas of exploitation [24]. However, avoiding intensive charcoal extraction is difficult if the demand for charcoal remains high, driving up prices and incentives to make charcoal [17]. It will require coordination at the provincial level of the charcoal licencing regime and forestry sectors, to ensure the frontier keeps moving away from Maputo City, or risk losing key ES in the long term.

### (c) Implications for livelihoods

Charcoal production is an important livelihood activity in our study area [25], and if charcoal production causes unsustainable loss of Mopane trees, local people could risk losing this livelihood activity in the long term. However, other studies have found a limited contribution of forests to rural livelihoods if other land uses, such as agriculture, can provide equal or higher livelihood benefits [53,54]. Agricultural land plays an important part in providing some natural resources [55], but this study found that several key provisioning services were specific to woodlands. A decline in woodland-specific services, such as firewood and construction materials, may cause appreciable welfare losses, especially for the most vulnerable [41]. Although other land covers such as agriculture are undoubtedly important for livelihoods, the loss or degradation of woodlands would still impact on essential services that cannot be provided from alternative land covers in our study area. In order to gain a full understanding of ES provision and impacts of changing woodlands, more studies on ES provision and livelihood benefits from other land cover types are required.

The primary benefit of charcoal for producers is for cash income generation, especially given a general lack of alternative income sources [56]. Charcoal production is one of the only ways to generate cash income in our study area, as alternatives such as cash crops are limited due to lack of access to markets and low productivity. Generally, income from charcoal is not enough to lift producers out of poverty [54], but it can mitigate the impacts of poverty in some instances [57,58], and provide an important safety net during hard times [41,56]. Thus, charcoal production may be beneficial to local populations if the costs of charcoal production impacts on natural resources do not outweigh the cash benefits to livelihoods. However, other studies conducted as part of the ACES project in our study area have found that the majority of the charcoal production income did not remain with local communities, and was largely exported by external large-scale producers, due to lack of support for community producers and poor governance in the forestry sector [25]. This pattern of benefit distribution disfavouring local village producers is common [59–61]. Therefore, evidence suggests that local people stand to lose their natural resources but do not gain the majority of the profits as a result of charcoal production in our study area [25]. Alternative livelihoods, improving governance in the forestry sector and supporting locally accountable management initiatives are key to maintaining important ES and improving benefits received from charcoal production for local populations, and are a key challenge in southern Mozambique.

## 5. Conclusion

This study finds that charcoal production is most likely to trade-off with firewood and woody construction materials in the Mopane woodlands of southern Mozambique, and declines in these services have already been occurring in villages with longer histories of charcoal production. However, even under very intense selective charcoal production scenarios in future, services were unlikely to disappear altogether. Some villages with lower-quality woodlands may be more vulnerable to further impacts of charcoal production, and should be prioritized in any management efforts.

This study contributes towards a better understanding of the ecological processes that govern ES provision and trade-offs in African woodlands, which can contribute towards managing woodlands for multiple ES. However, further work is required on Mopane re-growth rates in the context of sustainable charcoal production if suitable management options are to be recommended.

To minimize further trade-offs of charcoal production in the study area, charcoal production needs to remain highly selective in the species and size of trees extracted for charcoal and avoid further intensification of charcoal production. A switch to a ‘take anything’ harvesting regime risks losing key ES provision in the long term. To avoid increased intensification, the charcoal frontier must continue to expand to new areas of exploitation and allow for regeneration of woodlands to occur. To avoid further intensification of charcoal production and increase the cash benefits received by charcoal producers, improved governance in the forestry sector, coordination at the provincial level of the charcoal licencing regime, and support for local management initiatives are key challenges to overcome.

## Supplementary Material

Supplementary Materials

## Data accessibility

Data used in this article were collected as part of the ACES project. Data and protocols can be accessed by personal request to the lead author. Further contact information for the ACES project is available at https://miomboaces.wordpress.com. The dataset on species uses and local to species names supporting this article have been uploaded as part of the electronic supplementary material. The open source R program for statistical computing is available at https://www.r-project.org.
