# Supplementary material for: Charcoal production in the Mopane woodlands of Mozambique: what are the trade-offs with other ecosystem services?
Source: Philos Trans R Soc Lond B Biol Sci. 2016 Sep 19;371(1703):20150315. doi: 10.1098/rstb.2015.0315 (PMC4978873; doi:10.1098/rstb.2015.0315)
Supplement: Supplementary Materials [file rstb20150315supp1.pdf]

Supplementary material for Woollen, E. Ryan, C.M. Baumert, S. Vollmer, F. Fisher, J. Grundy, I. Fernando, J. Luz, A. Ribeiro, N. Lisboa, S.N. 2016. Charcoal production in the Mopane woodlands of Mozambique: what are the tradeoffs with other ecosystem services?, *Phil. Trans. R. Soc. B.* doi: 10.1098/rstb.2015.0315

Supplementary materials

Estimating stem diameters-at-breast-height (1.3 m)

We used a correction function to estimate the diameter-of-breast-height of stems not measured at 1.3 m from the ground. If the point of measurement on a stem was < 1.3 m, it has important implications for biomass estimation using allometric equations, as it will inflate the biomass values. The function used to estimate DBH at 1.3 m was based on tree taper measurements taken on thirty typical miombo wood tree species in central Mozambique (Casey Ryan, pers. comm.). The function used was:

$$d_{bh} = d_m - \left(k(130 - p)\right)$$

$$k = \frac{d_m - (0.8548 * d_m - 2.086)}{130 - p}$$

$$p < 130$$

Where the estimated diameter at breast-height (d_{bh}) is equal to the measured diameter (d_m) minus the product of k and the difference between breast-height and the actual height of measurement (p, cm). k is a constant calculated using the measured diameter (d_m) and the height of measurement (p, cm). The height of measurement must be < 130 cm (i.e. less than breast-height).

Ecological production functions

The ecological production functions used in this study to estimate the supply of provisioning ecosystem services (ES) from woodlands for each study village included charcoal, firewood, woody construction materials, food from trees, medicines derived from trees, and grass. All services were related to woodland structure so that the effects of changing woodland structure on ES provision could be modelled under different charcoal scenarios. The scenarios included a ‘no charcoal’ scenario, where all the tree stems suitable for charcoal (as described in eq. 1.1 below) were modelled as intact, and a ‘ total charcoal’ scenario, where all tree stems suitable for charcoal were modelled as cut. These scenarios could then be compared to the current estimates of ES provision from woodlands to assess the likely effects of charcoal production in the past and the future on ES provision at the village scale. Full details of ecological production functions are described below, and tables of the calculated parameters used in functions are available in Table S3.

Charcoal production involves the removal of woody biomass from woodlands, which is then pyrolysed in a low oxygen environment in a kiln to produce charcoal. The quality of charcoal is dependent on the quality of the wood (density and size of the woody stems), the kiln type, and the skill of the charcoal maker. Charcoal makers will maximise profits by maximising the quality of the charcoal. In our study site, traditional earthen kilns are used to produce charcoal, as other technologies are not available. Therefore, charcoal producers in our study area can only increase the quality of the charcoal by selecting high quality woody biomass for their kilns, and packing it correctly. The production function for woodlands to provide suitable woody biomass for charcoal is therefore a function of the availability of suitable tree stem sizes and species for charcoal. The production function can be described by:

$$E_{charcoal,i,s} = \left(\frac{\sum_{k=1}^{nplot,i} \frac{B_{charcoal\ i,k,s}}{B_{total\ i,k,s}}}{n_{plot,i,s}}\right)\left(\sum_{j=1}^{npix,i} B_{radar\ i,j}\right) AS_s \qquad \text{(Eq. 1)}$$

$$B_{charcoal\ i,k,s} = B \begin{cases} spp = \{TableS1\} \\ dbh > 14.0 \\ status = alive, not\ cut, not\ broken \end{cases} \quad \text{(Eq. 1.1)}$$

$$B_{total\ i,k,s} = B \begin{cases} dbh \geq 5.0 \\ status = \ not\ cut \end{cases} \quad \text{(Eq. 1.2)}$$

$$A = 0.0225 \quad \text{(Eq. 1.3)}$$

$$S_s = \frac{B_{total,i,s}}{B_{total,i,c}} \quad \text{(Eq. 1.4)}$$

where the potential provision of charcoal in land cover *i* and scenario *s* ($E_{charcoal\ i,s}$, Mg C) is the product of the mean fraction of total above-ground woody biomass that is suitable for charcoal in all plots *k* in land cover *i* and scenario *s* ($n_{plot,i,s}$), and the total sum of above-ground biomass for all radar pixels *j* in land cover *i* (*npix,i*), scaled to the hectare by the pixel size (*A*, ha) and the scenario using a radar scaling factor for scenario *s* (S_s). Suitable above-ground biomass for charcoal production for each plot *k* in land cover *i* and scenario *s* ($B_{charcoal\ i,k,s}$ Mg C ha^{-1}) was assessed as all stems of those tree species known to be used for charcoal production in the study area (Table S1), with a DBH > 14 cm, that were not cut, dead or broken. We used a DBH >14 to include only large stems of charcoal trees, and large stems were determined from biophysical data as the median diameter of all measured cut stems of *C. mopane* trees (Table S2). Total above-ground biomass for each plot *k* in land cover *i* and scenario *s* ($B_{total\ i,k,s}$ Mg C ha^{-1}) was assessed as all woody stems with a DBH > 5 cm that were not cut. The radar scaling factor for scenario *s* (S_s) was determined as the fraction of total above-ground biomass for land cover *i* and scenario *s* ($B_{total,i,s}$) divided by the total above-ground biomass for land cover *i* in the current scenario *c* ($B_{total,i,c}$) (i.e. change in total AGB between the current measured state and the modelled scenario).

Firewood could be defined as any woody biomass that will burn. However, from our social datasets, preferences for particular firewood species were found to exist in the study area. Therefore, firewood was defined as any standing woody biomass and any coarse woody debris of suitable species for burning (Table S1). We include standing woody biomass, as the household survey found that some households would cut live standing trees for firewood, at least on occasion. Thereby, the production function for firewood can be described by:

$$E_{firewood,i,s} = \left(\frac{\sum_{k=1}^{nplot,i} C_{firewood\ i,k,s}}{n_{plot,i,s}}\right) H_i + \left(\frac{\sum_{k=1}^{nplot,i} \frac{B_{firewood\ i,k,s}}{B_{total\ i,k,s}}}{n_{plot,i,s}}\right) \left(\sum_{j=1}^{npix,i} B_{radar\ i,j}\right) AS_s \quad \text{(Eq. 2)}$$

$$C_{firewood\ i,k,s} = B_{firewood\ i,k,s} * 0.05 \quad \text{(Eq. 2.1)}$$

$$B_{firewood\ i,k,s} = B \begin{cases} spp = TableS1 \\ dbh\ \geq 5 \\ status = not\ cut \end{cases} \quad \text{(Eq. 2.2)}$$

$$B_{total\ i,k,s} = B \begin{cases} dbh\ \geq 5 \\ status = not\ cut \end{cases} \quad \text{(Eq. 2.3)}$$

where the potential provision of firewood in land cover *i* and scenario *s* ($E_{firewood\ i,s}$ Mg C) is the mean biomass of coarse woody debris suitable for firewood ($C_{firewood\ i,k,s}$ Mg C ha^{-1}) for all plots *k* in land cover *i* and scenario *s* ($n_{plot,I,s}$) scaled to the total area of land cover *i* (H_i, ha), plus the product of the mean fraction of total above-ground woody biomass that is suitable for firewood in all plots *k* in land cover *i* and scenario *s* ($n_{plot,i,s}$), and the total sum of above-ground biomass for all radar pixels *j* in land cover *i* (*npix,i*), scaled to the hectare by the pixel size (*A*, ha) and the scenario using a radar scaling

factor for scenario *s* (S_s). Suitable coarse woody debris for firewood in plot *k* in land cover *i* and scenario *s* ($C_{firewood\ i,k,s}$) was estimated as 5 % of the above-ground woody biomass suitable for firewood ($B_{firewood\ i,k,s}$). 5 % was used as the plot data found the overall fraction of coarse woody debris that was of suitable species for firewood, to the total standing woody biomass of suitable species, was 0.05. Suitable above-ground woody biomass for firewood in plot *k* in land cover *i* and scenario *s* ($B_{firewood\ i,k,s}$) was assessed as all standing stems that were of species known to be used for firewood in the study area (Table S1) with a DBH > 5 cm and that were not cut. Total above-ground biomass for each plot *k* in land cover *i* and scenario *s* ($B_{total\ i,k,s}$ Mg C ha^{-1}) was assessed as all the woody stems with a DBH > 5cm that were not cut. The radar scaling factor for scenario *s* (S_s) was determined as in equation 1.4 above.

Woody construction materials are broadly defined in this study as any above-ground woody biomass collected from live trees used to construct houses, furniture, tools, or fences. Suitable woody biomass for construction will vary depending on its end use, where differing characteristics of wood may be preferred for different purposes. Therefore, we assume that tree species used for construction can be used as a proxy for suitable properties of the wood for construction purposes. Thereby, the production function can be described by:

$$E_{construction,i,s} = \left(\frac{\sum_{k=1}^{nplot,i} \frac{B_{construction\ i,k,s}}{B_{total\ i,k,s}}}{n_{plot,i,s}} \right) \left(\sum_{j=1}^{npix,i} B_{radar\ i,j} \right) A S_s \qquad \text{(Eq. 3)}$$

$$B_{construction\ i,k,s} = B \begin{cases} spp = \{Table\ S1\} \\ dbh \ \geq 5 \\ status = alive, not\ cut, not\ broken \end{cases} \qquad \text{(Eq. 3.1)}$$

$$B_{total\ i,k,s} = B \begin{cases} dbh \geq 5 \\ status = \ not\ cut \end{cases} \qquad \text{(Eq. 3.2)}$$

where the potential provision of construction material in land cover *i* and scenario *s* ($E_{constructionl\ i,s}$ Mg C) is the product of the mean fraction of total above-ground woody biomass that is suitable for construction in all plots *k* in land cover *i* and scenario *s* ($n_{plot,i,s}$), the total sum of above-ground biomass for all radar pixels *j* in land cover *i* (*npix,i*), scaled to the hectare by the pixel size (*A*, ha) and the scenario using a radar scaling factor for scenario *s* (S_s). Suitable biomass for construction in plot *k* in land cover *i* and scenario *s* ($B_{construction\ i,k,s}$) was assessed as all stems that were of species known to be used for construction purposes in the study area (Table S1) with a DBH >5 cm and that were not cut, broken or dead. Total above-ground biomass for each plot *k* in land cover *i* and scenarios *s* ($B_{total\ i,k,s}$ Mg C ha^{-1}) was assessed as all the woody stems with a DBH > 5cm that were not cut. The radar scaling factor for scenario *s* (S_s) was determined as in equation 1.4 above.

Food from woodlands can include fruits, mushrooms, insects, roots, leaves, honey and more. In this study we restrict our definition of food from woodlands to include those directly produced by trees in order to relate to woodland structure. We include only those edible insects which have a dependence on a particular tree species (e.g. Mopane worms). Therefore, the presence of tree species (i.e. number of stems) which can produce food will increase the woodland potential for providing food services. Thereby, the production function can be described by:

$$E_{food,i,s} = \left(\frac{\sum_{k=1}^{nplot,i} S_{food\ i,k,s}}{n_{plot,i,s}} \right) H_i \qquad \text{(Eq. 4)}$$

$$S_{food\ i,k,s} = S \begin{cases} spp = \{Table\ S1\} \\ dbh\ \geq 5 \\ status = alive, not\ cut \end{cases} \qquad \text{(Eq. 4.1)}$$

where the potential provision of food for consumption in land cover *i* and scenarios s ($E_{food\ i,s}$,total tree stems) is the product of the mean stem density of species that can produce food ($S_{food\ i,k,s}$ stems ha^{-1}) for all plots *k* in land cover *i* and scenario *s* ($n_{plot,i,s}$), scaled to the total area of land cover *i* (H_i, ha). Trees that can produce food in plot *k* in land cover *i* and scenario *s* ($S_{food\ i,k,s}$) were assessed as those tree stems of species known to produce food (fruits, roots, leaves, insects) in the study area (Table S1) with a DBH > 5 cm that were not dead or cut.

Medicinal plants are here defined as medicines or remedies which are derived from local tree materials, used in the maintenance of health as well as the prevention, diagnosis, improvement or treatment of physical and mental illness. Therefore, a woodland that has a greater presence of trees (i.e. number of tree stems) that could be used for medicinal purposes would have a higher potential to provide those medicines. Thereby, the production functions for medicines provided by trees would be:

$$E_{medicine,i,s} = \left(\frac{\sum_{k=1}^{nplot,i} S_{medicine\ i,k,s}}{n_{plot,i,s}}\right) H_i \qquad \text{(Eq. 5)}$$

$$S_{medicine\ i,k,s} = S \begin{cases} spp = \{Table\ S1\} \\ dbh \geq 5 \\ status = alive, not\ cut \end{cases} \qquad \text{(Eq. 5.1)}$$

where the potential provision of traditional medicines in land cover *i* and scenarios *s* ($E_{medicine,i,s}$ total tree stems) is the product of the mean stem density of species that can be used as medicines ($S_{medicine\ i,k,s}$ stems ha^{-1}) for all plots *k* in land cover *i* and scenarios *s* ($n_{plot,i}$), scaled to the total area of land cover *i* (H_i, ha). Trees that can provide medicines for plot *k* in land cover *i* and scenarios s ($S_{medicine}$) were assessed as those tree stems of species known to be used for medicine in the study area (Table S1) with a DBH > 5 cm that were not dead or cut.

Grass is used for several services in our study area including for roof thatch, livestock grazing and medicinal purposes. Due to the lack of data on grass species and their uses, we include grass biomass as a proxy for services related to grasses, where we assume greater grass biomass will provide more of these services to local communities. Grass biomass is highly variable in woodlands due to local scale factors such as fire occurrence, canopy cover, and other edaphic factors. Therefore, we estimate the maximum potential for grass biomass using a relationship found between measured plot level dry grass biomass and stem density (Fig. S1). A negative exponential quantile regression at the 90th percentile was fitted to the data using the package quantreg in *R* to model the maximum potential changes in grass biomass given changes in stem density. Thereby, the production function for grass biomass maximum potential provision is described by:

$$E_{grass,i,s} = \left(\frac{\sum_{k=1}^{nplot,i} G_{\ i,k,s}}{n_{plot,i,s}}\right) H_i \qquad \text{(Eq. 6)}$$

$$G_{i,k,s} = 3.45\ e^{-0.001 x_s} \qquad \text{(Eq. 6.1)}$$

where the maximum potential provision of dry grass biomass in land cover *i* and scenario *s* ($E_{grass\ i,s}$ Mg) is the mean estimated maximum potential of grass biomass ($G_{i,k,s}$ Mg ha^{-1}) for all plots *k* in land cover *i* and scenario *s* ($n_{plot,i,s}$) scaled to the total area of land cover *i* (H_i, ha). Plot estimated maximum

potential for dry grass biomass for plot *k* in land cover *i* and scenario *s* ($G_{i,k,s}$) is a function of stem density in scenario *s* (x_s, stems ha^{-1}).

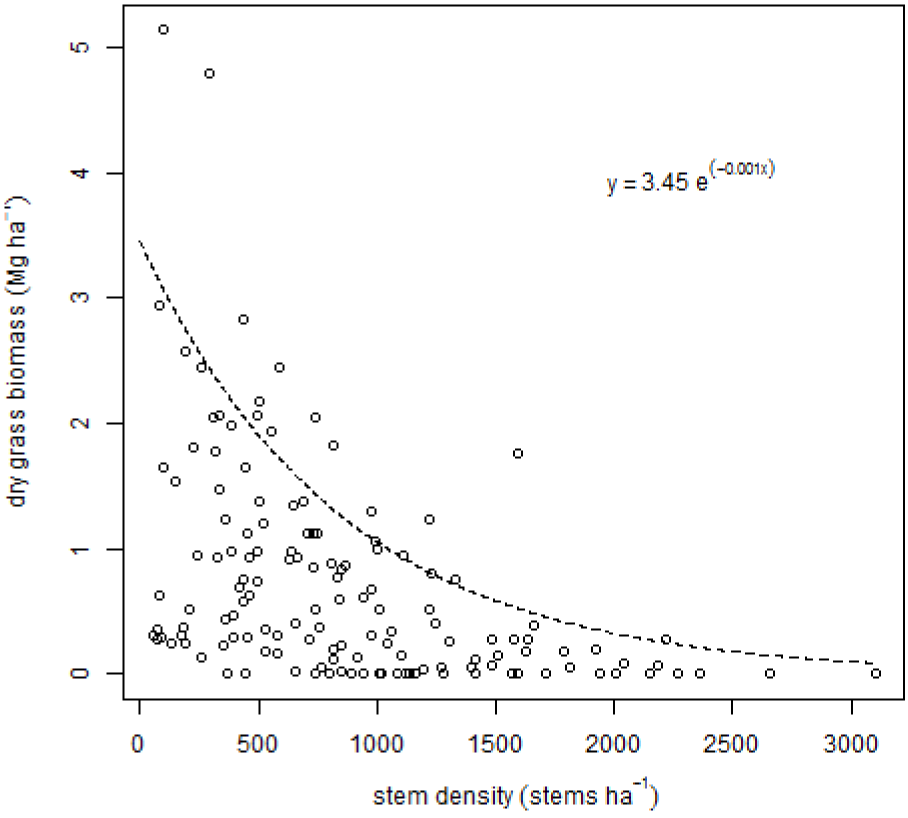

Fig. S1: Quantile regression at the 90th percentile of dry grass biomass as a function of stem density for all unburnt plots.

Total village ES potential; Using the production functions on each land cover type found in the village sample areas, and in each scenario, we can estimate the total potential ES provision at village scales as follows:

$$E_{x,y,s} = \sum_{i=1}^{nLC\ y} E_{x,i,s} \quad \text{(Eq. 7)}$$

where the total estimated potential provision of ES service *x* in village *y* and scenario *s* is the sum of potential provision of service *x* (one of charcoal, firewood, construction, food, medicine, grass) in each land cover *i* and scenario *s*. The number of land covers (*nLC*) is determined by those land covers found to occur within village *y* sample area.

Woodland types

In the study area, five different woodland types were identified. These woodland types were distinct, and are depicted below for visual comparisons (Fig. S2). The woodland types identified included Androstachys forest (picture A), Combretum woodland (picture B), Mopane woodland (picture C), Boscia woodland (picture D) and shrub Mopane (picture E). Androstachys forest was almost exclusively *Androstachys johnsonii* tree species with no grass cover, where other tree species occurred in clearings, on termite mounds, or on edges of these distinct forest patches. Combretum woodland and Mopane woodland had more open canopies and greater grass cover. Mopane woodland was dominated by *Colophospermum mopane* species, whereas Combretum woodland had a more diverse species assemblage. Boscia woodland was also more open with some grass cover, but trees tended to be small and therefore with less biomass. Shrub Mopane was unique in that the

Mopane trees were all below 2 m tall, and there were Aloe species present. Shrub Mopane also had very little grass cover, often occurring clumped together.

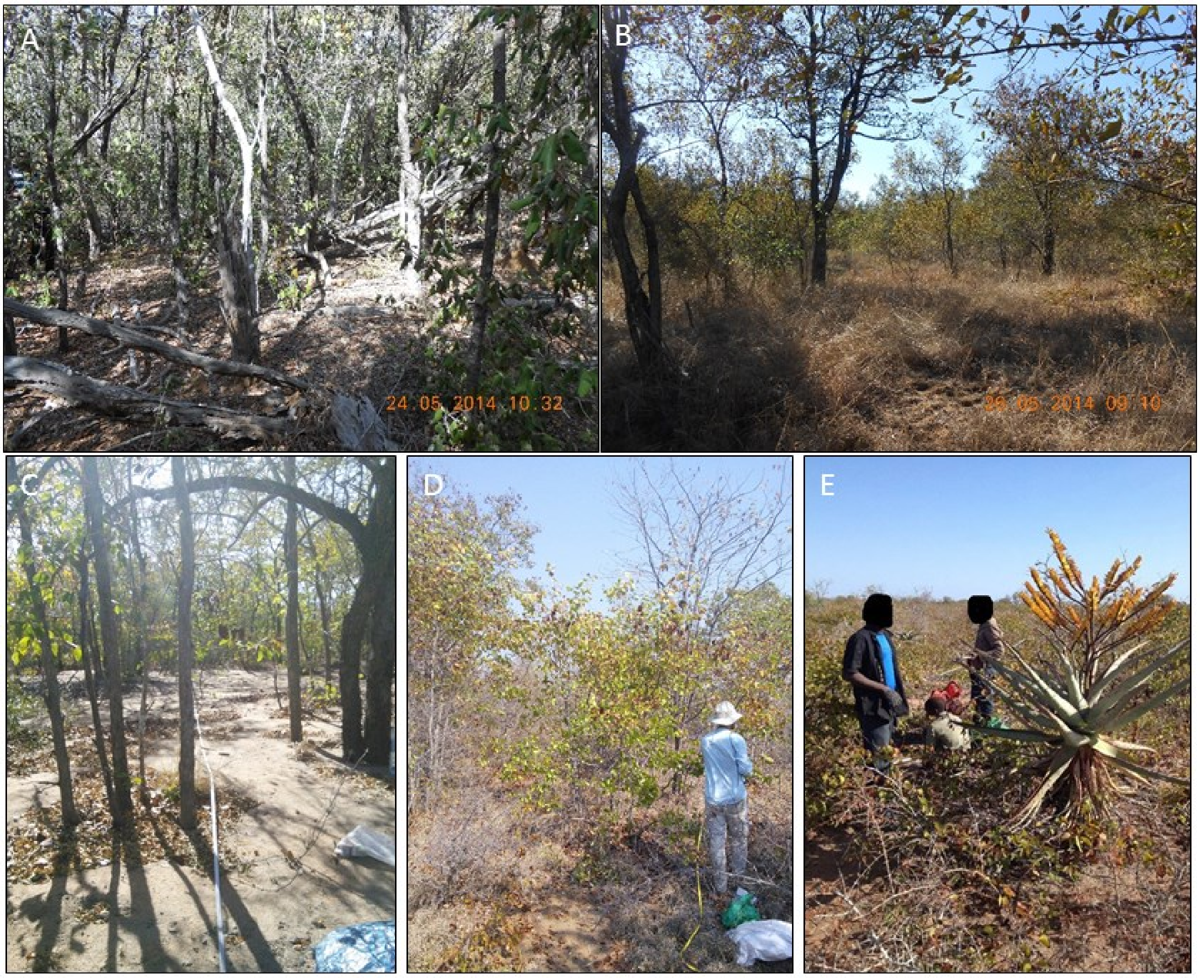

Fig S2: Photos depicting the five different woodlands types found in the study area, A) Androstachys forest, B) Combretum woodland, C) Mopane woodland, D) Boscia woodland and, C) Shrub Mopane. Photos have been anonymised where necessary. Photos courtesy of Emily Woollen and Casey Ryan.

Supplementary material for Woollen, E. Ryan, C.M. Baumert, S. Vollmer, F. Fisher, J. Grundy, I. Fernando, J. Luz, A. Ribeiro, N. Lisboa, S.N. 2016. Charcoal production in the Mopane woodlands of Mozambique: what are the tradeoffs with other ecosystem services?, *Phil. Trans. R. Soc. B.* doi: 10.1098/rstb.2015.0315

Supplementary tables and figures

Table S1: All local tree species names recorded in the study area, their scientific names, and their known uses. Uses include provisioning ES of charcoal production, firewood, woody construction materials, food and medicinal plants. Blank cells indicate when there was no record of the species being used for the specific services. The local language is Shangaan.

**Local name**	**Species**	**Ecosystem service**				
		**Charcoal**	**Firewood**	**Construction**	**Food**	**Medicine**
incaia	*Acacia burkei*					+
sessane	*Acacia grandicornuta*					
caia	*Acacia nigrescens*	+				
changua	*Acacia nilotica kraussiana*					
ximua	*Adenium multiflorum*					
chamfuta	*Afzelia quanzensis*			+		+
linhane	*Albizia brevifolia*					
nala	*Albizia petersiana petersiana*					
mbesso	*Albizia versicolor*					+
aloe	*Aloe marlothii orientalis*					
simbiri	*Androstachys johnsonii*			+		+
tita	*Artabotrys brachypetalus*				+	+
nulu	*Balanites maughamii*				+	+
nhiye	*Berchemia discolor*				+	
xicutse	*Boscia albitrunca*			+	+	+
ximafamafane	*Boscia mossambicensis*					
xigatlho	*Capparis erythrocarpos rosea*					
khau	*Capparis tomentosa*				+	
chanatse	*Colophospermum mopane*	+	+	+	+	+
chigugutzo	*Combretum molle*					+
fambanibolili	*Combretum spp.*					
fucane	*Combretum spp.*					
xivondzone	*Combretum spp.*	+	+			+
khoro	*Commiphora africana africana*					
chifata	*Commiphora pyracanthoides pyracanthoides*					
nhasimbitsane	*Croton pseudopulchellus*					+
chilutse	*Dalbergia melanoxylon*					+
ndzengua	*Dichrostachys cinerea*					+
chitomatomane	*Diospyros loureiriana loureiriana*			+	+	
ntoma	*Diospyros mespiliformis*			+	+	
chire	*Diospyros natalensis natalensis*			+	+	

tsupa	*Diospyros spp.*					
xipongodze	*Diospyros spp.*			+		
lhangula	*Euclea natalensis natalensis*				+	
xitsalala	*Gardenia spp.*					
tambacorota	*Gardenia volkensii volkensii volkensii*					
chacuare	*Glenniea africana*					+
sihana	*Grewia bicolor*					
sipane	*Grewia spp.*					+
tsotso	*Guibourtia conjugata*	+	+			+
cungunuti-munti	*Hugonia orientalis*					+
chibalekela	*Hymenocardia ulmoides*					
tsica	*Hymenodictyon parvifolium*					+
vumahila	*Kirkia acuminata*					
xibombocanhe	*Lannea schweinfurthii*					
nhatsuluane	*Limeum fenestratum*					+
thanthangate	*Maerua nervosa*		+			+
wambo	*Manilkara mochisia*				+	+
mbova	*Monodora junodii junodii*					
fumutso	*Newtonia hildebrandtii hildebrandtii*					+
nhamutane	*Olax dissitiflora*					
chinungunafi	*Ozoroa obovata obovata*					
cuacuashi	*Pappea capensis*					
bandzo	*Philenoptera violacea*					+
ndzari	*Ptaeroxylon obliquum*					
tsandzadlhopfo	*Pterocarpus lucens antunesii*					
phesua	*Rhoicissus revoilii*					
poco	*Salvadora persica persica*					+
canhe	*Sclerocarya birrea caffra*				+	+
chilati	*Spirostachys africana*					+
utsulo	*Strophanthus petersianus*					+
chicuacuacuane	*Strychnos decussata*					+
cuacua	*Strychnos madagascariensis*			+	+	+
massala	*Strychnos spinosa*				+	+
malambacupica	*Suregada zanzibariensis*				+	
chachandau	*Terminalia prunioides*					
tsemawate	*Terminalia prunioides*					
conola	*Terminalia sericea*					+
tchequelenhane	*Thilachium africanum*					+
filwa	*Vangueria infausta infausta*				+	+

munwane	*Vernonia amygdalina*					
chifutumba	*Vitex harveyana*				+	
dzungua	*Xeroderris stuhlmannii*	+			+	+
balatangate	*unknown1*					
ndzangalangoha	*unknown10*					
nhabzane	*unknown12*					
nhafembafembane	*unknown13*					
nhambrecuane	*unknown14*					+
ntamba	*unknown15*					
pafa	*unknown17*					
phandamangela	*unknown18*					
phassamala	*unknown19*					+
ganicomo	*unknown2*		+			+
semane	*unknown20*			+		
senguelete	*unknown21*				+	+
tlhavelankuzi	*unknown23*					
tsetsenho	*unknown24*					
tsoqueta	*unknown25*					
vulaussico	*unknown26*					
xibandwane	*unknown27*					
xicuavutlhulo	*unknown28*					
ximamarucua	*unknown29*					
lhambandzaca	*unknown3*					
ximuwamawava	*unknown30*					
xitswa	*unknown31*				+	
xivatlhancombe	*unknown32*					
xilangamalho	*unknown33*					
lisotchua	*unknown4*					
lumanhama	*unknown5*	+				+
madlhane	*unknown6*					
mbela	*unknown8*				+	
nambuadji	*unknown9*					
	**Total species**	**6**	**5**	**10**	**21**	**39**

Table S2: Summary of all measured cut stems in all plots (n = 154) across all villages. The number of measured cut stems (n) and the mean, median, minimum and maximum estimated diameter-at-breast-height (DBH) at 1.3 m are shown for each species.

**Local name**	**Species**	**n**	**Mean DBH (cm)**	**Med DBH (cm)**	**Min DBH (cm)**	**Max DBH (cm)**
chanatse	*Colophospermum mopane*	360	15.5	14.0	5.0	44.1
simbiri	*Androstachys johnsonii*	174	9.2	7.3	5.0	50.1
xivondzone	*Combretum spp.*	76	12.8	11.7	5.1	35.7
xicutse	*Boscia albitrunca*	58	16.1	13.3	5.0	54.3
chilati	*Spirostachys africana*	38	13.9	12.6	7.5	27.8
tsotso	*Guibourtia conjugata*	10	8.9	8.7	5.0	12.8
incaia	*Acacia burkei*	5	9.9	9.9	5.6	15.9
conola	*Terminalia sericea*	4	9.0	8.6	5.9	12.9
fucane	*Combretum spp.*	2	5.5	5.5	5.4	5.6
bandzo	*Philenoptera violacea*	1	14.0	14.0	14.0	14.0
caia	*Acacia nigrescens*	1	25.6	25.6	25.6	25.6
chifata	*Commiphora pyracanthoides*	1	27.8	27.8	27.8	27.8
lumanhama	*unknown5*	1	18.4	18.4	18.4	18.4
tsetsenho	*unknown24*	1	6.7	6.7	6.7	6.7
wambo	*Manilkara mochisia*	1	7.1	7.1	7.1	7.1
xilangamalho	*unknown33*	1	5.0	5.0	5.0	5.0

Table S3: Ecological production function parameters calculated from forest plot data for each gradient, woodland type and scenario. Parameters were calculated within gradient classes, rather than for individual villages, to increase the sample size *n*. Villages within each gradient are post-boom (villages A-C), boom (villages D-E), and pre-boom (village F-G). The number of plots (*n*), and the mean calculated production function parameter and the standard error of the means are shown. Standard errors could not be calculated when number of plots (*n*) was less than 4. For 95 % confidence intervals on estimates, multiply standard error of the mean by 2.

	**Parameter**	***n***	**Charcoal**	**Firewood (CWD)**	**Firewood (trees)**	**Construction**	**Food**	**Medicine**	**Grass**	**Radar scaling factor**
	**Function**	$n_{plot,i,s}$	$\frac{\sum_{k=1}^{nplot,i} \frac{B_{charcoal\ i,k,s}}{B_{total\ i,k,s}}}{n_{plot,i,s}}$	$\frac{\sum_{k=1}^{nplot,i} C_{firewood\ i,k,s}}{n_{plot,i,s}}$	$\frac{\sum_{k=1}^{nplot,i} \frac{B_{firewood\ i,k,s}}{B_{total\ i,k,s}}}{n_{plot,i,s}}$	$\frac{\sum_{k=1}^{nplot,i} \frac{B_{construction\ i,k,s}}{B_{total\ i,k,s}}}{n_{plot,i,s}}$	$\frac{\sum_{k=1}^{nplot,i} S_{food\ i,k,s}}{n_{plot,i,s}}$	$\frac{\sum_{k=1}^{nplot,i} S_{medicine\ i,k,s}}{n_{plot,i,s}}$	$\frac{\sum_{k=1}^{nplot,i} G_{i,k,s}}{n_{plot,i,s}}$	S_s
	**Units**	count	Fraction	Mg C ha^{-1}	Fraction	Fraction	Stems ha^{-1}	Stems ha^{-1}	Mg ha^{-1}	Fraction
	Scenario	*Current*	*Current*	*Current*	*Current*	*Current*	*Current*	*Current*	*Current*	*Current*
**Gradient**	**Woodland type**									
**Post-boom**	Androstachys	0								
	Mopane	33	0.15 ± 0.04	0.26 ± 0.05	0.60 ± 0.06	0.58 ± 0.05	617 ± 62	772 ± 74	1.60 ± 0.13	1
	Combretum	17	0.26 ± 0.05	0.3 ± 0.04	0.48 ± 0.09	0.02 ± 0.01	64 ± 22	547 ± 75	1.97 ± 0.15	1
	Boscia	13	0	0	0	0.77 ± 0.08	518 ± 82	533 ± 75	1.99 ± 0.15	1
	Shrub Mopane	3	0	0	0.01	0.04	77	77	3.12	1
**Boom**	Androstachys	11	0.07 ± 0.04	0.06 ± 0.02	0.03 ± 0.01	0.66 ± 0.07	8 ± 4	1538 ± 151	0.82 ± 0.12	1
	Mopane	2	0.52	1.01	0.92	0.92	973	989	1.82	1
	Combretum	29	0.31 ± 0.04	0.31 ± 0.12	0.41 ± 0.05	0.14 ± 0.03	128 ± 23	403 ± 43	2.19 ± 0.11	1
	Boscia	0								
	Shrub Mopane	0								
**Pre-boom**	Androstachys	13	0.03 ± 0.01	0.07 ± 0.03	0.05 ± 0.02	0.64 ± 0.04	76 ± 42	1937 ± 174	0.54 ± 0.08	1
	Mopane	16	0.38 ± 0.05	0.41 ± 0.11	0.69 ± 0.06	0.63 ± 0.06	365 ± 44	482 ± 51	2.05 ± 0.13	1
	Combretum	17	0.15 ± 0.04	0.32 ± 0.07	0.42 ± 0.07	0.13 ± 0.03	92 ± 19	774 ± 108	1.5 ± 0.18	1
	Boscia	0								
	Shrub Mopane	0								

Table S3 continued: 'No charcoal production' scenario 1

	**Parameter**	***n***	**Charcoal**	**Firewood (CWD)**	**Firewood (trees)**	**Construction**	**Food**	**Medicine**	**Grass**	**Radar scaling factor**
	**Function**	$n_{plot,i,s}$	$\frac{\sum_{k=1}^{nplot,i} \frac{B_{charcoal\ i,k,s}}{B_{total\ i,k,s}}}{n_{plot,i,s}}$	$\frac{\sum_{k=1}^{nplot,i} C_{firewood\ i,k,s}}{n_{plot,i,s}}$	$\frac{\sum_{k=1}^{nplot,i} \frac{B_{firewood\ i,k,s}}{B_{total\ i,k,s}}}{n_{plot,i,s}}$	$\frac{\sum_{k=1}^{nplot,i} \frac{B_{construction\ i,k,s}}{B_{total\ i,k,s}}}{n_{plot,i,s}}$	$\frac{\sum_{k=1}^{nplot,i} S_{food\ i,k,s}}{n_{plot,i,s}}$	$\frac{\sum_{k=1}^{nplot,i} S_{medicine\ i,k,s}}{n_{plot,i,s}}$	$\frac{\sum_{k=1}^{nplot,i} G_{\ i,k,s}}{n_{plot,i,s}}$	S_s
	**Units**	count	Fraction	Mg C ha^{-1}	Fraction	Fraction	Stems ha^{-1}	Stems ha^{-1}	Mg ha^{-1}	Fraction
	Scenario	*No charcoal*	*No charcoal*	*No charcoal*	*No charcoal*	*No charcoal*	*No charcoal*	*No charcoal*	*No charcoal*	*No charcoal*
**Gradient**	**Woodland type**									
Post-boom	Androstachys	0								
	Mopane	33	0.29 ± 0.05	0.38 ± 0.05	0.66 ± 0.05	0.66 ± 0.05	685 ± 61	843 ± 71	1.48 ± 0.12	1.21
	Combretum	17	0.34 ± 0.06	0.38 ± 0.05	0.54 ± 0.08	0.02 ± 0.01	64 ± 22	624 ± 82	1.83 ± 0.15	1.10
	Boscia	13	0	0	0	0.77 ± 0.08	518 ± 82	533 ± 75	1.99 ± 0.15	1.00
	Shrub Mopane	3	0	0	0.01	0.04	77	77	3.12	1.00
Boom	Androstachys	11	0.07 ± 0.04	0.06 ± 0.02	0.03 ± 0.01	0.66 ± 0.07	8 ± 4	1541 ± 152	0.82 ± 0.12	1.00
	Mopane	2	0.52	1.04	0.92	0.93	979	995	1.81	1.03
	Combretum	29	0.34 ± 0.05	0.31 ± 0.12	0.41 ± 0.05	0.14 ± 0.03	128 ± 23	432 ± 45	2.13 ± 0.11	1.01
	Boscia	0								
	Shrub Mopane	0								
Pre-boom	Androstachys	13	0.04 ± 0.02	0.07 ± 0.03	0.05 ± 0.02	0.64 ± 0.04	76 ± 42	1950 ± 173	0.53 ± 0.08	1.00
	Mopane	16	0.57 ± 0.05	0.63 ± 0.11	0.77 ± 0.05	0.77 ± 0.05	443 ± 47	570 ± 51	1.87 ± 0.12	1.35
	Combretum	17	0.2 ± 0.04	0.32 ± 0.07	0.42 ± 0.07	0.13 ± 0.03	92 ± 19	851 ± 123	1.42 ± 0.19	1.00
	Boscia	0								
	Shrub Mopane	0								

Table S3 continued: 'Total charcoal production' scenario 2

	**Parameter**	***n***	**Charcoal**	**Firewood**		**Construction**	**Food**	**Medicine**	**Grass**	**Radar scaling factor**
	**Function**	$n_{plot,i,s}$	$\frac{\sum_{k=1}^{nplot,i}\frac{B_{charcoal\ i,k,s}}{B_{total\ i,k,s}}}{n_{plot,i,s}}$	$\frac{\sum_{k=1}^{nplot,i}C_{firewood\ i,k,s}}{n_{plot,i,s}}$	$\frac{\sum_{k=1}^{nplot,i}\frac{B_{firewood\ i,k,s}}{B_{total\ i,k,s}}}{n_{plot,i,s}}$	$\frac{\sum_{k=1}^{nplot,i}\frac{B_{construction\ i,k,s}}{B_{total\ i,k,s}}}{n_{plot,i,s}}$	$\frac{\sum_{k=1}^{nplot,i}S_{food\ i,k,s}}{n_{plot,i,s}}$	$\frac{\sum_{k=1}^{nplot,i}S_{medicine\ i,k,s}}{n_{plot,i,s}}$	$\frac{\sum_{k=1}^{nplot,i}G_{\ i,k,s}}{n_{plot,i,s}}$	S_s
	**Units**	count	Fraction	Mg C ha^{-1}	Fraction	Fraction	Stems ha^{-1}	Stems ha^{-1}	Mg ha^{-1}	Fraction
	Scenario	*Total charcoal*	*Total charcoal*	*Total charcoal*	*Total charcoal*	*Total charcoal*	*Total charcoal*	*Total charcoal*	*Total charcoal*	*Total charcoal*
**Gradient**	**Woodland type**									
Post-boom	Androstachys	0								
	Mopane	33	0	0.13 ± 0.01	0.53 ± 0.06	0.52 ± 0.06	591 ± 59	744 ± 71	0.48 ± 0.10	0.77
	Combretum	17	0	0.15 ± 0.03	0.42 ± 0.09	0.04 ± 0.01	58 ± 21	493 ± 71	1.11 ± 0.19	0.76
	Boscia	13	0	0	0	0.77 ± 0.08	518 ± 82	533 ± 75	0.79 ± 0.22	1.00
	Shrub Mopane	3	0	0	0.01	0.04	77	77	0.34	1.00
Boom	Androstachys	11	0	0.02 ± 0.01	0.01 ± 0.01	0.71 ± 0.06	5 ± 3	1527 ± 151	0.02 ± 0.01	0.99
	Mopane	2	0	0.29	0.71	0.71	928	944	2.67	0.98
	Combretum	29	0	0.09 ± 0.01	0.35 ± 0.05	0.22 ± 0.05	118 ± 22	359 ± 40	1.32 ± 0.22	0.88
	Boscia	0								
	Shrub Mopane	0								
Pre-boom	Androstachys	13	0	0.02 ± 0.01	0.02 ± 0.01	0.67 ± 0.03	76 ± 42	1920 ± 173	0.09 ± 0.04	0.97
	Mopane	16	0	0.12 ± 0.02	0.53 ± 0.08	0.49 ± 0.07	310 ± 41	425 ± 46	0.76 ± 0.18	0.53
	Combretum	17	0	0.17 ± 0.03	0.34 ± 0.06	0.17 ± 0.04	92 ± 19	734 ± 100	0.65 ± 0.18	0.74
	Boscia	0								
	Shrub Mopane	0								

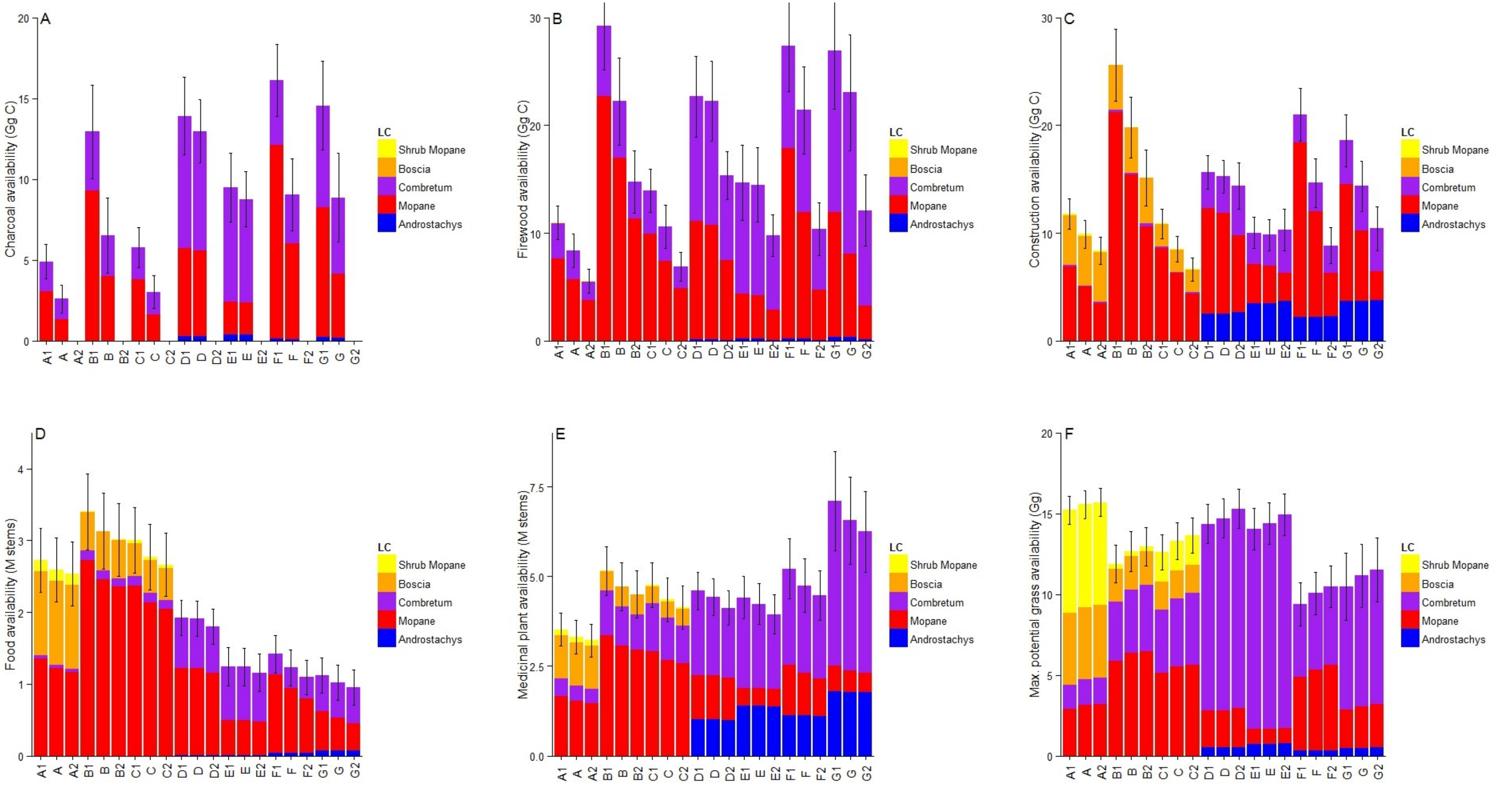

Fig. S3: Changes to ecosystem service availability (in absolute terms) for A) charcoal, B) firewood, C) woody constriction materials, D) food from woodlands, E) medicinal plants, and F) maximum potential for grass under different charcoal scenarios for each of seven villages in Mabalane District. The 'no charcoal' scenario (indicated by the number 1 after each village letter on the x-axis) models all charcoal trees as intact, as if they had never been cut. The 'total charcoal' scenario (indicated by the number 2 after each village letter on the x-axis) models all charcoal trees as cut and removed from the woodlands. Current ES availabilities are shown in bars where no number follows village letters on the x-axis. Error bars are 95% confidence intervals.
